# Downregulation of miR-506-3p Facilitates EGFR-TKI Resistance through Induction of Sonic Hedgehog Signaling in Non-Small-Cell Lung Cancer Cell Lines

**DOI:** 10.3390/ijms21239307

**Published:** 2020-12-06

**Authors:** Inamul Haque, Hameem I. Kawsar, Hannah Motes, Mukut Sharma, Snigdha Banerjee, Sushanta K. Banerjee, Andrew K. Godwin, Chao H. Huang

**Affiliations:** 1 Cancer Research Unit, Veterans Affairs Medical Center, Kansas City, MO 64128, USA; sa199469@atsu.edu (H.M.); sbanerjee@kumc.edu (S.B.); sbanerjee2@kumc.edu (S.K.B.); 2 Department of Pathology and Laboratory Medicine, University of Kansas Medical Center, Kansas City, KS 66160, USA; agodwin@kumc.edu; 3 Division of Medical Oncology, University of Kansas Medical Center, Kansas City, KS 66160, USA; skawsar@kumc.edu; 4 Kirksville College of Osteopathic Medicine, Andrew Taylor Still University, Jefferson St, Kirksville, MO 63501, USA; 5 Research Service, Veterans Affairs Medical Center, Kansas City, MO 64128, USA; mukut.sharma@va.gov

**Keywords:** EGFR, tyrosine kinase inhibitor (TKI), resistance, non-small-cell lung cancer (NSCLC), Sonic Hedgehog, miR-506-3p

## Abstract

Non-small-cell lung cancer (NSCLC) patients with epidermal growth factor receptor (EGFR) mutation eventually develop resistance to EGFR-targeted tyrosine kinase inhibitors (TKIs). Treatment resistance remains the primary obstacle to the successful treatment of NSCLC. Although drug resistance mechanisms have been studied extensively in NSCLC, the regulation of these mechanisms has not been completely understood. Recently, increasing numbers of microRNAs (miRNAs) are implicated in EGFR-TKI resistance, indicating that miRNAs may serve as novel targets and may hold promise as predictive biomarkers for anti-EGFR therapy. MicroRNA-506 (miR-506) has been identified as a tumor suppressor in many cancers, including lung cancer; however, the role of miR-506 in lung cancer chemoresistance has not yet been addressed. Here we report that miR-506-3p expression was markedly reduced in erlotinib-resistant (ER) cells. We identified Sonic Hedgehog (SHH) as a novel target of miR-506-3p, aberrantly activated in ER cells. The ectopic overexpression of miR-506-3p in ER cells downregulates SHH signaling, increases E-cadherin expression, and inhibits the expression of vimentin, thus counteracting the epithelial–mesenchymal transition (EMT)-mediated chemoresistance. Our results advanced our understanding of the molecular mechanisms underlying EGFR-TKI resistance and indicated that the miR-506/SHH axis might represent a novel therapeutic target for future EGFR mutated lung cancer treatment.

## 1. Introduction

Lung cancer comprises approximately 13% of all cancer diagnoses. However, it is the leading cause of cancer-related death worldwide [1]. In the US, it is estimated that 228,820 new cases will be diagnosed in 2020, with the estimated death of 135,20 patients [2]. Lung cancer is divided into two groups: small-cell lung cancer and non-small lung cancer (NSCLC). NSCLC has four major histopathologic groups: squamous, adenocarcinoma, adenosquamous and large cell. Adenocarcinoma accounts for the majority of NSCLC. While significant progress has been made over the past decade, lung cancer prognosis is still bleak, with only 19% of patients surviving more than five years [3]. In the United States, the overall 5 year relative survival rate for NSCLC was only 25% in 2015 [4]. With the increased screening of high-risk patients, modern diagnostic tools, and the development of new treatment modalities, including targeted therapies and immunotherapies, the mortality from lung cancer is declining. However, approximately 40% of NSCL are diagnosed at stage IV, and consequently have a poor prognosis and overall survival [5]. There has been significant progress in the treatment of advanced-stage NSCLC, including the discovery of EGFR and BRAF mutation, anaplastic lymphoma kinase (ALK) gene rearrangement, ROS1 fusion gene, among others that allows the use of tyrosine kinase inhibitors (TKI) specific for these actionable gene mutations/rearrangement. The majority of NSCLC cancer harboring actionable EGFR mutations occurs in adenocarcinoma histology. They can be treated with EGFR-specific TKIs, such as erlotinib, gefitinib, afatinib, dacomitinib, or osimertinib, as frontline therapy. However, patients will invariably develop mutations conferring resistance to these TKIs. Although the majority resistance is related to the development of gatekeeper p.T790M mutation and amplification of the c-MET, other mechanisms of resistance to these TKI remains poorly understood. 

Recently, miRNAs have been investigated as potential biomarkers for diagnosis and predictors of prognosis for NSCLC [6]. Prognostic value of specific miRNAs has been reported, with most focus on miRNA present in the small extracellular vesicles (sEVs), frequently referred to as exosomes in plasma and serum [7]. sEVs can activate signaling pathways or deliver nucleic acids to distant cells. Among these miRNAs, miR-506 has been shown to play a crucial role in various cancers, including lung cancer [8,9,10,11,12,13,14,15]. miR-506 is involved in cell proliferation, differentiation, migration, and invasion [16]. It can act as a tumor suppressor gene in malignant bronchial epithelial cells, and restoration of miR-506 can suppress tumor growth in vitro and in vivo [17]. Additional research also showed that miR-506 prevented TGFβ-induced EMT, increased E-cadherin expression, and inhibited migration and invasion by targeting SNAIL family zinc finger-2 [18]. The Sonic Hedgehog (SHH) pathway, which is involved in organogenesis and embryogenesis, is regulated by miRNAs [19,20]. Abnormal activation of the SHH pathway can lead to the genesis of an aggressive form of basal cell carcinoma called ‘Gorlin’s syndrome.’ The SHH pathway is also abnormally activated in medulloblastoma and breast cancer [21,22]. Dysregulation of the SHH pathway can lead to increased intracellular β-catenin and hence an increase in invasion and metastatic potential [23]. Recent studies have shown that the SHH pathway is upregulated in a subset of lung adenocarcinoma with EGFR mutation [24,25], and inhibition of the SHH pathway can also enhance the activity of EGFR inhibitor gefitinib in pancreatic cancer cells [26]. Inhibition of Hedgehog signaling has been reported to sensitize NSCLC cells to EGFR-TKIs [20].

Given the importance of unique cross-talks between miR-506-3p, EGFR, and SHH, we sought to explore the role of SHH signaling in miR-506-3p-mediated EGFR-TKI resistance in NSCLC EGFR mutant cells.

## 2. Results

### 2.1. EGFR-TKI-Resistant Cells Demonstrate Cancer Stem Cell Behavior and Resist Death in Response to Erlotinib Treatment

**Figure 1:**
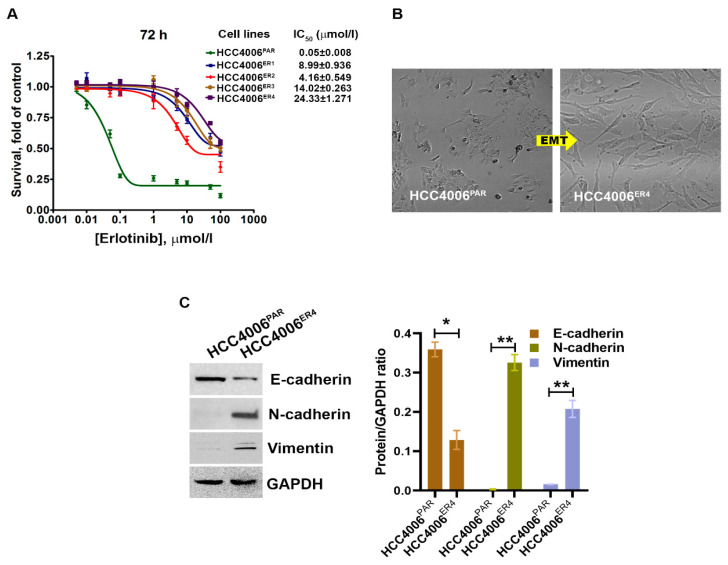
The changes in cell survival and epithelial–mesenchymal transition in NSCLC cells by erlotinib. (**A**) The survival curves of erlotinib-sensitive (HCC4006^PAR^) and erlotinib-resistant cells (HCC4006^ER4^) in the presence of different concentrations of erlotinib. (**B**) The photomicrographs represent the morphological changes (from epithelial-mesenchymal transition, EMT) in HCC4006^PAR^ and HCC4006^ER4^ cells (Magnification, 10×). (**C**) Western blot analyses of EMT markers in HCC4006^PAR^ and HCC4006^ER4^ cells. The right panel shows the semi-quantitative estimation by densitometry analysis of protein bands. For semi-quantitative analysis, E-cadherins, N-cadherins, and vimentin bands are evaluated upon normalization with the corresponding housekeeping GAPDH protein band. Data are expressed as the mean ± SD. * *p* < 0.001, ** *p* < 0.0005 when compared with HCC4006^PAR^ cells.

Erlotinib-sensitive parental NSCLC cells (HCC4006^PAR^) and four different erlotinib-resistant clones, HCC4006^ER1^, HCC4006^ER2^, HCC4006^ER3^ and HCC4006^ER4^, were incubated with different concentrations of erlotinib up to 72 h. Cell survival was assessed at 72 h. The survival of parental cells, HCC4006^PAR^, was significantly reduced in a dose-dependent manner (Figure 1A). The IC*_50_* value for erlotinib in the parental cell lines was 0.05 ± 0.008 μmol/L. However, erlotinib-resistant cell lines showed significantly decreased sensitivity to erlotinib with the IC_50_ values > 100 folds than the parental cells. These results indicate that all resistant clones are significantly resistant to erlotinib-induced cell death.

Cancer stem cells (CSCs), which are the outcome of epithelial–mesenchymal transition (EMT) and the hallmark of aggressive phenotypes, have been reported to promote drug resistance [27]. Thus, we investigated the cancer stemness by determining the morphology and some EMT markers. We observed extensive morphological changes in the resistant cells as compared to parental cells. These included loss of intracellular connections and loss of polarity, which are the critical features of mesenchymal cells (Figure 1B). Further, the evaluation of EMT markers in resistant and parental cells using Western blotting showed that erlotinib-sensitive cells exhibit increased E-cadherin expression, which is an epithelial cell marker. In contrast, HCC4006^ER4^ showed increased N-cadherin expression and vimentin expression, which are common mesenchymal cell markers (Figure 1C). This result indicates that erlotinib-resistant cells, HCC4006^ER4^ display mesenchymal cells’ properties, both phenotypically and by expression of mesenchymal cell-specific proteins.

### 2.2. EGFR-TKI-Resistant Cells Show a Significant Increase in Migration and Colony Formation Compared to Non-Resistant Cells

**Figure 2:**
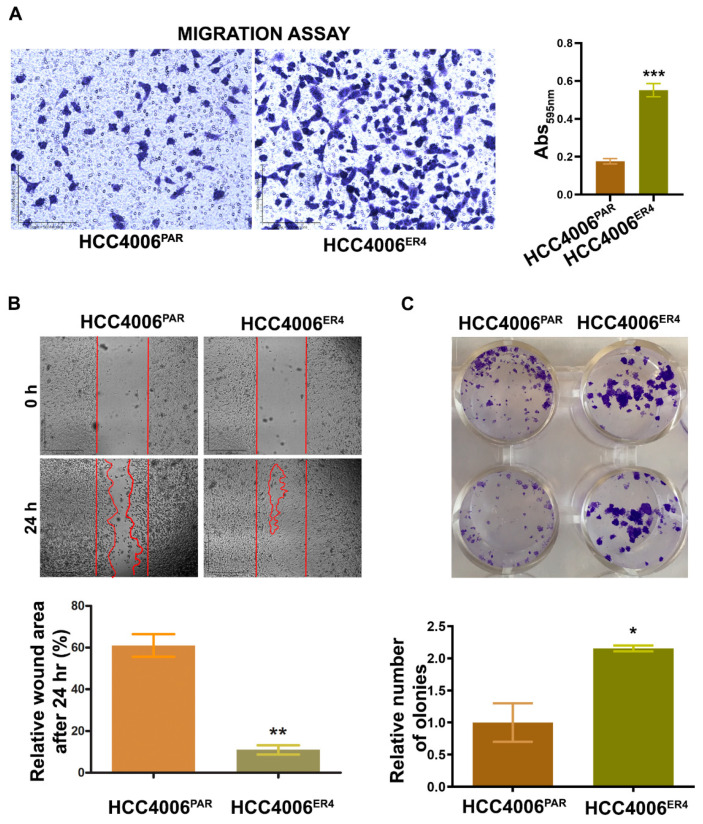
Erlotinib resistance-induced migratory and colony-forming potential of parental and resistant cells. (**A**) The migratory potential of HCC4006^PAR^ and HCC4006^ER4^ cells was determined by the transwell migration assay. The photomicrographs represent the difference in the in vitro migration of HCC4006^PAR^ and HCC4006^ER4^ cells toward the serum for 24 h (Magnification, 10×) histogram showing crystal violet absorbance at 595 nm. Values in the bar graphs represent the mean ± SD (*n* = 6). *** *p* < 0.0001 compared to HCC4006^PAR^ cells. (**B**) Representative phase-contrast images of scratch-wound healing exhibit the motility of HCC4006^PAR^ and HCC4006^ER4^ cells. Cell motility into the wound area was examined and measured by microscopy, and the photomicrograph was taken at 0 h and 24 h (Magnification, 2×). Lower panel shows a bar graph illustrating percentage wound area at indicated time points during the scratch wound assay (** *p* < 0.005 vs. HCC4006^PAR^). (**C**) HCC4006^PAR^, and HCC4006^ER4^ cells were allowed to grow for 10 days and colony formation was visualized by staining with crystal violet. The image represents the best of the replicates (*n* = 3). Colonies were counted by the Colony Doc-It imaging station using Colony Doc-It imaging software. The bar graph shows the relative number of colonies. Data are expressed as the mean ± SD, * *p* < 0.03.

Migratory behavior is the hallmark of aggressive cancers and necessary for the metastatic spread and growth in distant organs. Our next objective was to investigate whether erlotinib-resistant cells can migrate faster than the non-resistant cells. The in vitro migration studies found that erlotinib-resistant cells showed significantly increased migration than parental cells (Figure 2A).

Next, to investigate the motility of erlotinib-resistant cells, we carried out wound healing assays. Both parental and resistant cells were grown to near confluency in separate dishes and wounded with a sterile pipette tip. Cell migration and wound healing were assessed after 24 h. While parental cells showed cell migration and partial wound healing, erlotinib-resistant cells showed complete healing of the wound with complete confluency (Figure 2B). These results indicate that the erlotinib-resistant cells can move and grow faster compared to parental cells.

Next, we investigated the cell viability by determining the anchorage-dependent colony-forming ability of both parental cells and resistant cells. We found that erlotinib-resistant cells have significantly increased number/larger colony-forming ability (Figure 2C) compared to parental cells, a characteristic feature of aggressive cancer.

### 2.3. EGFR-TKI-Resistant Cells Showed Downregulation of miR-506-3p and Upregulation of Its Target SHH and Glioma-Associated Oncogene Homolog Zinc Finger Protein 1 (GLI1] Expression

**Figure 3:**
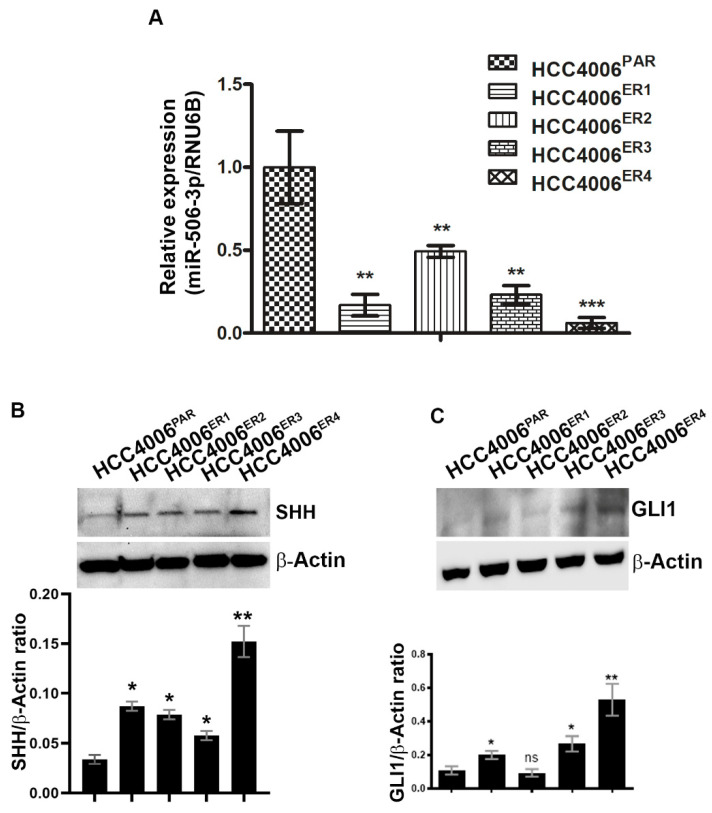
Downregulation of miR-506-3p and upregulation of its target Sonic Hedgehog (SHH) and glioma-associated oncogene homolog zinc finger protein 1 (GLI1) expression. (**A**) The bar graph indicates the quantitative relative expression of miR-506-3p in erlotinib-sensitive cells (HCC4006^PAR^) and erlotinib-resistant cells (HCC4006^ER1, ER3 and ER4)^ by real-time PCR. Data are expressed as the mean ± SD, ** *p* < 0.005, *** *p* < 0.001 when compared to HCC4006^PAR^. (**B**) Western blot analysis and quantification (bar graph) of SHH protein erlotinib sensitive and resistant cells. β-actin is used as a loading control. Data show the mean ± SD and are representative of at least three independent experiments. * *p* < 0.01, ** *p* < 0.003 when compared to HCC4006^PAR^. (**C**) Detection and quantification (bar graph) of GLI1 protein level in sensitive and resistant cells using Western blot analysis. β-actin is used as a loading control. Data are expressed as the mean ± SD ns, non-significant and * *p* < 0.01, ** *p* < 0.007 when compared to HCC4006^PAR^.

miRNAs have recently been extensively studied and implicated in tumorigenesis in multiple cancers, including nasopharyngeal cancer [28], ovarian cancer [29], cervical cancer [30], and hematological malignancies [15] among others. We investigated the expression of miR-506-3p in both parental and resistant cells by extracting total RNA and quantification by quantitative real-time PCR (qRT-PCR). Erlotinib-resistant clones, HCC4006^ER4^ showed significantly decreased expression of miR-506-3p compared to parental cells (Figure 3A). We further investigated SHH and GLI1 expression in parental and erlotinib-resistant cells by using SDS-PAGE and immunoblotting. Both SHH and GLI1 had increased expression in erlotinib-resistant cells compared to parental cells (Figure 3B,C). These findings suggest that underexpression of miR-506-3p (in erlotinib-resistant cells) may be linked with increased expression of SHH and GLI1. Both SHH and GLI1 have been reported to be overexpressed in gastric, bile duct, lung, and kidney cancers, among others [31,32,33,34,35,36].

### 2.4. miR-506-3p Overexpression Sensitizes EGFR-TKI-Resistant Cells to Erlotinib-Induced Cell Death, Induced Mesenchymal–Epithelial Transition (MET), Downregulates SHH Expression and Stemness

**Figure 4:**
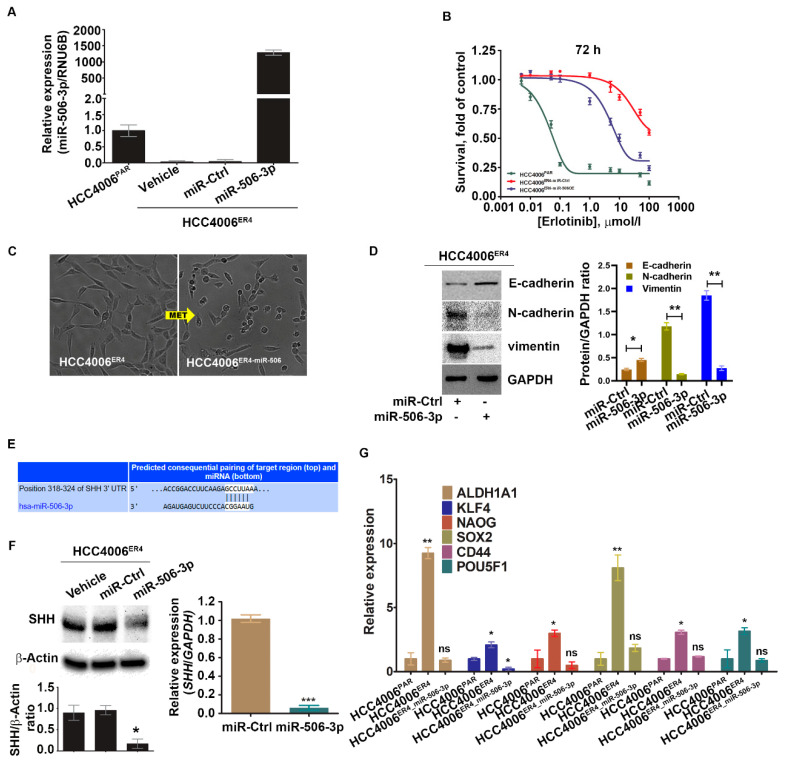
Ectopic expression of miR-506-3p increases erlotinib sensitivity by reversing EMT and downregulating SHH and other stem cell markers. (**A**) Real-time PCR analysis of miR-506-3p after transfection with miR-506-3p vectors into HCC4006^ER4^ cells. The expression of miR-506-3p was normalized to the expression in HCC4006^PAR^ cells. Data are expressed as the mean ± SD, * *p* < 0.005, ** *p* < 0.0002 when compared to HCC4006^PAR^. (**B**) The survival curves of HCC4006^PAR^, miR-control erlotinib-resistant cells (HCC4006^ER4-miR-Ctrl^), and miR-506-3p overexpressed resistant cells (HCC4006^miR-506-3pOE^) in the presence of different concentrations of erlotinib. (**C**) The photomicrographs represent the morphological changes (from mesenchymal-epithelial transition, MET) in HCC4006^ER4^ and HCC4006^miR-506-3pOE^ cells (Magnification, 10×). (**D**) Western blot analyses of EMT markers in HCC4006^ER4^ cells transfected with miR-Ctrl and miR-506-3p vectors. The right panel shows the SDi-quantitative estimation by densitometry analysis of protein bands. For semi-quantitative analysis, E-cadherins, N-cadherins, and vimentin bands are evaluated upon normalization with the corresponding housekeeping GAPDH protein band. * *p* < 0.005, ** *p* < 0.0035 when compared with HCC4006^ER4-miR-Ctrl^ cells. (**E**) Seed matched sequences for miR-506-3p in the coding region of *SHH* mRNA were identified by TargetScan 7.2. The sequences in white are the locations of the potential seed-matched sequences for the 3’UTR *SHH* and miR-506-3p. (**F**) Reduced expression of SHH in HCC4006^miR-506-3pOE^ cells compared to erlotinib-resistant cells (ER4-vehicle and ER4-miR-control). The bar graph shows the relative protein expression; β-actin was used as a loading control. Right panel indicates the quantitative relative expression of *SHH* mRNA in resistant cell lines transfected with miR-Ctrl and miR-506-3p vectors by real-time PCR. Data are presented as the mean ± SD for three independent experiments. Data are expressed as the mean ± SD * *p* < 0.001, *** *p* < 0.0001. (**G**) Real-time PCR analysis of stemness markers in HCC4006^PAR^, HCC4006^ER4^, and HCC4006^miR-506-3pOE^ cells. ns, non-significant; * *p* < 0.01, ** *p* < 0.001 vs. HCC4006^PAR^.

We investigated the implication of overexpression of miR-506-3p in erlotinib-resistant cells (which has low miR-506-3p expression). Erlotinib-resistant cells, HCC4006^ER4^,were transfected with pCMV-miR-506-3p vector or empty vector without the miR-506-3p gene (purchased from Origene, USA; cat. no. SC400433), and the expression level of miR-506-3p was quantified by qRT-PCR (Figure 4A). The resultant miR-506-3p-overexpressing cells, HCC4006^ER4-miR-506-3pOE^, were used to evaluate the sensitivity to erlotinib by crystal violet assay compared with HCC4006ER4-miRNA control (HCC4006^ER4-miR-Ctrl^). HCC4006^ER4-miR-506-3pOE^ cells showed increased erlotinib sensitivity and diminished survival compared to control (Figure 4B). Microscopic examination of these cells showed HCC4006^ER4^ cells with a mesenchymal-like phenotype, whereas HCC4006^ER4-miR-506-3pOE^ cells displayed an epithelial-like phenotype, demonstrating MET (Figure 4C). We then investigated the EMT markers in miR-506-3p-overexpressing cells. We found that E-cadherin expression was significantly increased, whereas vimentin and N-cadherin expression was decreased in HCC4006^ER4-miR-506-3pOE^ cells compared to HCC4006^ER4-miR-Ctrl^ cells (Figure 4D). In an effort to identify the putative target of miR-506-3p, we used TargetScan software (TargetScan 7.2 to predict its complementary binding targets. Interestingly, we found that SHH is one of the predicted targets of miR-506-3p (Figure 4E). We also showed that overexpression of miR-506-3p in erlotinib-resistant cells resulted in the downregulation of SHH protein and mRNA level (Figure 4F). Next, we studied the stemness of HCC4006^PAR^ cells, erlotinib-resistant cells (HCC4006^ER4^), and HCC4006^ER4-miR-506-3pOE^. Total RNA was extracted from cells, and relative quantification of specific stem cell markers, ALDH1A1, KLF4, Nanog, SOX2, CD44, and POU5F1, were evaluated by qRT-PCR (Figure 4G). Overexpression of miR-506-3p resulted in the reversal of stem cell markers to a comparable level of HCC4006^PAR^. Taken together, these findings suggest that overexpression of miR-506-3p re-sensitizes erlotinib-resistant cells to erlotinib reprograms MET, downregulates expression of SHH protein, and stemness, all of which are associated with reduced growth and metastatic potential.

### 2.5. miR-506-3p Overexpression Inhibits Migration and Increases Apoptosis, Which Is Attenuated by SHH Ligand

**Figure 5:**
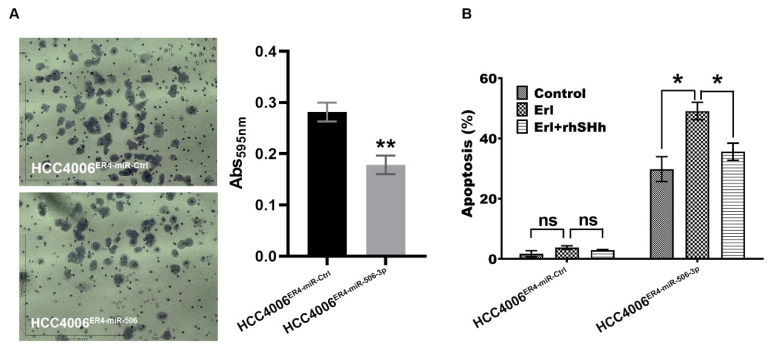
Migration and apoptotic activity of erlotinib-resistant cells and miR-506-3p-overexpressing erlotinib-resistant cells. (**A**) The migratory potential of HCC4006^ER4-miR-Ctrl^ and HCC4006^ER4-miR-506-3pOE^ cells was determined by the transwell migration assay. The photomicrographs represent the difference in the in vitro migration of HCC4006^ER4-miR-Ctrl^ and HCC4006^ER4-miR-506-3pOE^ cells toward the serum for 24 h (Magnification, 10×). Histogram showing crystal violet absorbance at 595 nm. Values in the bar graphs represent the mean ± SD (*n* = 6). ** *p* < 0.001 compared to HCC4006^ER-miR-Ctrl^ cells. (**B**) Overexpression of miR-506-3p vectors in HCC4006^ER4^ cells promoted apoptosis after exposure to erlotinib which is counteracted by treatment with both erlotinib and rhSHH protein. Data are expressed as the mean ± SD, ** *p* < 0.001.

We demonstrated that erlotinib-resistant cells have an increased ability to migrate, most likely via underexpression of miR-506-3p. To confirm this finding, we overexpressed miR-506-3p in erlotinib-resistant cells and conducted a migration assay. As anticipated, the overexpression of miR-506-3p in erlotinib-resistant cells showed decreased migration by approximately 37% (Figure 5A). We also conducted apoptosis assays using HCC004^ER4-miR-Ctrl^ and HCC4006^ER4-miR-506-3pOE^ cells. Erlotinib-resistant cells with overexpression of miR-506-3p showed increased apoptosis by 30% (Figure 5B). Since our data showed that miR-506-3p overexpression was associated with SHH expression (Figure 4F), we pre-treated HCC4006^ER4-miR-506-3pOE^ cells with rhSHH to investigate its effect on apoptosis. Pre-treatment of miR-506-3p-overexpressing cells with rhSHH demonstrated significant resistance (*p* < 0.005) to apoptosis (Figure 5B). Colletively, these findings confirm that erlotinib-resistant cells are more aggressive due to their increased migration potential and resistance to apoptosis via underexpression of miR-506-3p and activation of the SHH pathways.

### 2.6. SHH Reverses miR-506-3p Overexpression-Induced Erlotinib Sensitivity

**Figure 6:**
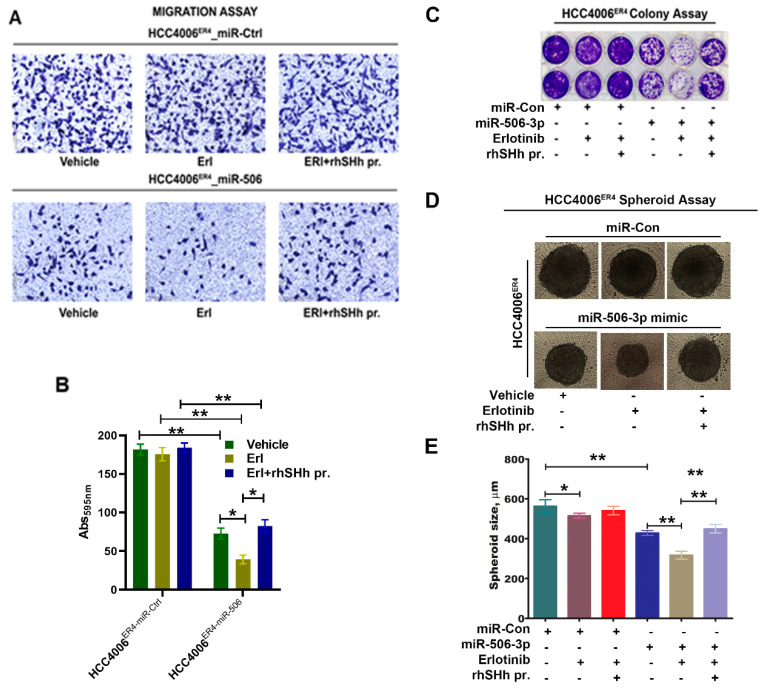
Effects of SHH ligand (rhSHH) on cell migration, colony formation, tumor spheroid formation, and spheroid size. (**A**) miR-Ctrl and miR-506-3p mimic transfected cells were exposed to vehicle, erlotinib, and rhSHH protein, and their migratory potential of was determined by transwell migration assay. (**B**) Histogram showing crystal violet absorbance at 595 nm. Values in the bar graphs represent the mean ± SD (*n* = 6). * *p* < 0.003, ** *p* < 0.0002 (**C**) miR-Ctrl and miR-506-3p mimic transfected cells were exposed to vehicle, erlotinib, and erlotinib-rhSHH protein combination, and allowed to grow for 10 days. Cell viability was analyzed by colony formation assay; cells were fixed in methanol and stained with crystal violet. (**D** and **E**) Single-cell suspensions from pretreated cells were re-suspended at a density of 500 cells/mL mammocult media in a 96-well spheroid microplate. The size of the spheroids in the specified experimental setup was monitored and recorded alternate days for 8–10 days. Data are expressed as the mean ± SD, * p < 0.02, ** p < 0.0002.

We demonstrated that miR-506-3p underexpression is associated with increased expression of SHH (Figure 3B) and is associated with increased invasiveness and EMT phenotype of lung cancer cells through the induction of the SHH signaling pathway. Moreover, ectopic expression of miR-506-3p in erlotinib-resistant cells inhibits SHH signaling. We further examined the effects of SHH ligand on miR-506-3p overexpressed cells and its response to erlotinib. HCC004^ER4-miR-Ctrl^ and HCC4006^ER4-miR-506-3pOE^ cells were treated with erlotinib and rhSHH. The migratory potential of treated and untreated cells was evaluated using Boyden migration chamber assays. HCC004^ER4-miR-Ctrl^ showed a similar rate of migration in the presence of either erlotinib or erlotinib and rhSHH protein (Figure 6A, upper panels, and Figure 6B, first three bars). However, HCC4006^ER4-miR-506-3pOE^ cells showed reduced migration in the presence of erlotinib (Figure 6A, lower panels, and Figure 6B, fifth bar). The reduced migration of erlotinib-treated cells was reversed when pretreated with rhSHH protein (Figure 6A, lower panels, and Figure 6B, sixth bar). Next, we studied the colony formation ability of these cells, and it was decreased in HCC4006^ER4-miR-506-3pOE^ cells in the presence of erlotinib. However, this effect was reversed when the SHH ligand, rhSHH, was added (Figure 6C). We then conducted a spheroid assay to measure the self-renewal and multipotent nature of HCC004^ER4-miR-Ctrl^ HCC4006^ER4-miR-506-3pOE^ cells in the presence of only erlotinib and both erlotinib and rhSHH protein. Single-cell suspensions from pretreated cells were re-suspended at a density of 500 cells/mL mammocult media in a 96-well spheroid microplate. The size of the spheroids was monitored and recorded on alternate days for 8–10 days. There was no significant change in the spheroid size of HCC004^ER4-miR-Ctrl^ cells in the presence of erlotinib and in the combination of erlotinib and rhSHH protein (Figure 6D,E). Treatment of HCC4006^ER4-miR-506-3pOE^ cells with erlotinib resulted in a smaller spheroid (26.2% inhibition, *p* < 0.0001), which was effectively reversed when both erlotinib and rhSHH protein was used (Figure 6D,E). These findings, taken together, suggest that SHH ligand (rhSHH protein) can effectively reverse the effects of miR-506-3p overexpression-induced erlotinib sensitivity.

## 3. Discussion

In current clinical practice, molecular testing using techniques, such as next-generation sequencing (NGS), polymerase chain reaction (PCR), and fluorescent in situ hybridization (FISH), are performed for advanced-stage NSCLC to identify actionable mutation or gene rearrangement/fusion. EGFR-TKI sensitizing mutations in exons 19 and 21 are found in 50% of Asian and 10% of Caucasian patients with NSCLC [37]. These mutations result in the constitutive kinase activation of the tyrosine kinase domain and activation of downstream signaling pathways, which induces cellular proliferation and increases survival. Patients harboring these mutations are usually treated with EGFR-specific TKIs, such as erlotinib, gefitinib, afatinib, dacomitinib, and osimertinib [33]. However, most patients with EGFR mutations develop resistance to first-generation TKIs (erlotinib, gefitinib) or second-generation TKI (afatinib) with the emergence of resistance mutations in EGFR (i.e., p.T790M) in approximately 60% of the patients [38], cMET alterations [39], or transformation to small-cell lung cancer (SCLC) [40]. Approximately 18% of cases, the mechanism of resistance is not defined. We investigated the role of miR-506 and the SHH pathway in the development of resistance to EGFR-TKI. Our study described a novel mechanism of EGFR resistance to EGFR-TKI through activation of the SHH pathway induced by underexpression of miR 506. This leads to an increase in migration, colony formation, and EMT. Our investigation showed that erlotinib-resistant cells (HCC4006^ER4^) showed a significant reduction in miR-506-3p expression (Figure 3A). This underexpression of miR-506-3p in erlotinib-resistant lung cancer cell lines corroborates with other published reports where downregulation of miR-506-3p expression is associated with poorly differentiated, aggressive tumors, with an increased tendency of distant metastasis and decreased survival [41,42]. In our experiments, erlotinib-resistant cells also demonstrated EMT, both phenotypically (Figure 1B) and by increased N-cadherin and vimentin expression, which are markers of mesenchymal cells and reduction in E-cadherin (Figure 1C). Transdifferentiation of erlotinib-sensitive parental cells from epithelial to erlotinib-resistant mesenchymal cells are marked by changes in phenotype and protein expression profile (Figure 1B,C). Erlotinib-resistant cells displayed mesenchymal phenotype and increased expression of N-cadherin and vimentin. The phenotypic expression of EMT has clinical implications as it is associated with reduced clinical activity of erlotinib with a lack of PFS and OS benefit in patients treated with erlotinib in the absence of E-cadherin expression [43]. 

Clinically, EGFR-TKI-resistant NSCLC follows an aggressive course with increased invasiveness and metastasis with poor outcomes. Our study showed that erlotinib-resistant cells showed significantly increased ability to migrate (Figure 2A), heal (Figure 2B), and to form a colony (Figure 2C), which are distinct characteristics of mesenchymal cells and aggressive tumors. Our findings in the above experiments mirror the clinical course of the disease, and this acquired aggressiveness of erlotinib-resistant cells appears to be, in conjunction with other mechanisms, mediated by EMT.

Hedgehog signaling is involved in early stages and metastatic tumors and is associated with increased metastatic potential and tissue invasion; inhibition of this pathway has been reported to reduce tumor cell proliferation [44,45,46,47,48]. It is estimated that the Hedgehog signaling pathway contributes to developing one-third of all cancers [49]. SHH ligands induce expression of one of its target genes, GLI genes, and the GLI proteins regulate the target gene(s) expression by binding with their promoters and promote tumorigenesis [50]. Our experiments showed that erlotinib-resistant cells underexpress miR-506-3p and overexpress SHH and GLI1 (Figure 3B,C), compared to erlotinib-sensitive parental cells. These findings indicate the activation of the SHH pathway, which is inversely related to miR-506-3p expression and has increased proliferation and migration potential. To further confirm our findings, we overexpressed miR-506-3p in erlotinib-resistant cells (HCC4006^ER4]^ and verified the overexpression by qRT-PCR (Figure 4A). This resistance can be reversed by overexpressing miR-506-3p in these resistant cells through reprogramming MET (Figure 4D). Overexpression of miR-506 was also reported to cause MET, suppression of adhesion, increased migration, and invasion in breast cancer cell lines [51]. 

The EMT leads to increased aggressiveness of the EGFR mutant lung cancer cells. This could be related to the increase proliferation of cancer stem cells. The presence of tumor stem cells has been reported in multiple cancers, including lung cancer with EGFR mutation [52]. We investigated the stemness of erlotinib-sensitive, erlotinib-resistant, and miR-506-3p-overexpressing resistant cells. Our results show that erlotinib-resistant cells have increased expression of stem cell markers, which was reversed in miR-506-3p-overexpressing cells (Figure 4G). This result indicates that erlotinib-resistant cells acquire stemness, enabling them to increase rapidly with appropriate stimulation. Typically, these stem cells within the tumor divide slowly [53], but can be stimulated to proliferate by appropriate signals, including Hedgehog signaling [54]. Overexpression of miR-506-3p reversed the cancer cells’ stemness, which reduced their proliferation potential, thereby resulting in less aggressive cancer. These findings are consistent with the literature, in which the inhibition of SHH is synergistic with EGFR inhibition in reducing the self-renewal of stem-like cells [55].

We also demonstrated that underexpression of miR-506-3p in erlotinib-resistant cells is associated with activation of the SHH signaling pathway (Figure 3B) and overexpression of miR-506-3p reduces SHH expression (Figure 4F). The effect of the activation of the SHH pathway was further studied by using SHH ligand in both erlotinib-resistant cells and erlotinib-resistant cells with ectopic overexpression of miR-506-3p. As expected, miR-506-3p-overexpressing erlotinib-resistant cells showed reduced migration response to Erlotinib (Figure 5A). However, when these cells were pretreated with rhSHH, it demonstrated significantly increased migration. This increase in migration in the presence of SHH ligand indicates that activation of the SHH signaling pathway reverses the effect of high miR-506-3p. 

To show the drug resistance, we also investigated the colony-forming ability of erlotinib-resistant cells with or without miR-506-3p mimic in erlotinib’s presence and absence with or without SHH ligand. Erlotinib-resistant cells with miR-506-3p did form only a few colonies in the absence of SHH activation with SHH ligand. However, there was a significantly increased colony formation when SHH ligand was added. This result indicates that activation of the SHH pathway with SHH ligand reversed the inhibitory effect of miR-506-3p in erlotinib’s presence. Activation of the SHH pathway with rhSHH also reversed the inhibitory effect of erlotinib on spheroid formation in the presence of erlotinib, and this difference was statistically significant (Figure 6C,D). These results also suggest that overexpression of miR-506-3p renders the erlotinib-resistant cells less able to form colonies. Endogenous miR-506 plays an essential role in cellular differentiation and tumorigenesis [56]. Recently, Guo et al. reported that miR-506miR-506-3p-3p is downregulated in cell lines and showed similar findings in 52 patients with advanced NSCLC. They also demonstrated that the expression of miR-506-3p was inversely related to the advanced stage and metastasis of NSCLC. Patients with NSCLC who had lower expression of miR-506-3p-3p had a poor prognosis than patients with higher expression [6]. Our findings can have clinical implications given recent advancements in therapeutic research in the miR-506-3p and SHH pathways in cancer treatment. Hedgehog pathway inhibitors (HPI), such as saridegib, glasdegib, soidegib, vismodegib and taladegib have been investigated and approved for the treatment of basal cell carcinoma [57,58]. Inhibition of GLI with anti-sense oligonucleotides reduced the growth rate and tumor size in murine intravesical orthotopic human bladder cancer model [59]. Recently, triptolide was shown to inhibit tumorosphere formation, reduced the stemness and tumorigenicity in lung cancer cells via inhibition of the SHH-GIL1 pathway [60]. Moreover, the therapeutic potential of miR-506-3p is also actively explored. Since miR-506-3p is underexpressed in cancer, tools to increase the levels of miR-506-3p in cancer cells have been explored. In one such experiment, miR-506-3p was incorporated in 1,2-Dioleoyl-sn-Glycero-3-Phosphatidylcholine (DOPC) nanoliposomes and delivered to ovarian cancer in a mouse model, which resulted in MET with increased E-cadherin expression and decreased tumor growth [18]. Systemic use of miR-506-3p sensitized ovarian cancer and showed the increased antitumor effect of platinum and olaparib in an animal model [61]. Further investigation is warranted to explore the use of novel drugs and methods targeting miR-506-3p expression and test the use of HPIs in NSCLC with EGFR mutation resistant to EGFR-TKIs.

**Figure 7:**
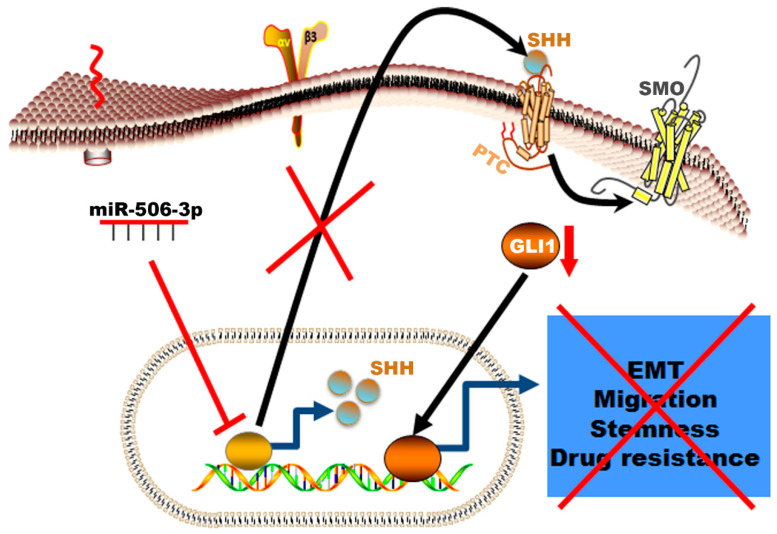
Schematic illustration of the mechanism by which miR-506-3p suppresses TKI resistance by targeting Sonic Hedgehog signaling in non-small-cell lung cancer with EGFR mutation. The Figure generated using Science slides software

In summary, our data showed a novel mechanism of resistance in erlotinib resistance of EGFR mutated NSCLC mediated by decreased expression of miR-506-3p and activation of SHH (Figure 7). The erlotinib-resistant cells are characterized by epithelial–mesenchymal transition, high proliferation rate, high migration rate, resistance to apoptosis, and increased colony formation ability. These phenotypic profiles indicate the aggressive nature of the erlotinib-resistant NSCLC cells and correlates with worse clinical course in patients who develop resistance while on EGFR-TKI therapy. This resistance can be reversed by increased expression of miR-506-3p, which reverses EMT to MET, increases sensitivity to apoptosis, decreases the cells’ stemness, and reduces proliferation. These reprogramming events are mediated via suppressing the SHH signaling pathway (Figure 7).

Further investigation is warranted to explore the use of novel drugs and methods targeting miR-506-3p expression and test the use of HPIs in NSCLC with EGFR mutation that are resistant to EGFR-TKIs.

## 4. Materials and Methods 

### 4.1. Generation of EGFR-TKI-Resistant Cell Lines 

EGFR-mutant human lung cancer cell line HCC4006 was obtained from ATCC (Manassas, VA, USA). The cells were cultured in RPMI-1640 growth medium supplemented with 10% fetal bovine serum (Gibco, USA) at 37 °C in a humidified 5% CO2 incubator. Erlotinib-resistant cells were generated as described previously [62] with minor modification. Briefly, HCC4006 cells were exposed chronically to increasing concentrations of erlotinib (Selleckchem, Houston, TX, USA) starting from 500 nmol/L to 20 µmol/L. The resultant clones were designated as HCC4006^ER1^ to HCC4006^ER4^, following expansion from a single cell. All the resistant clones were maintained in media containing 1 µmol/L erlotinib. Before any experiment, the cells were cultured in erlotinib free media for at least a week.

### 4.2. Cell Viability Assay 

To determine the effect of different concentrations of erlotinib on the viability of HCC4006 and HCC4006 ER cells, we performed crystal violet-based cell viability (Cell Biolabs, San Diego, CA, USA) in a 96-well format as previously described [63]. The cell viability was measured using iMARK^TM^ microplate absorbance reader (Bio-Rad, Hercules, CA, USA) at a wavelength of 595 nm.

### 4.3. Western Blot Analysis

The Western blot analysis was performed as we previously described [64]. Briefly, cells were washed with phosphate buffer saline (PBS), lysed in RIPA Buffer and centrifuged. Equal amounts of protein determined by the BCA method (Pierce BCA protein assay kit, Waltham, MA, USA) were subjected to SDS-PAGE and transferred onto nitrocellulose membranes (EMD Millipore, Burlington, MA, USA). Membranes were incubated overnight at 4 °C with specific antibodies followed by incubation with HRP-conjugated anti-rabbit (cat. no. 7074) or anti-mouse (cat. no. 7076) secondary antibody (1:10,000 dilution; Cell Signaling Technology, Inc.) for 30 min at room temperature. Signals were established using a chemiluminescence detection kit (Pierce ECL Western blotting substrate, Thermo Scientific, Rockford, IL, USA). Band intensities were quantitated using the program ImageJ (NIH) [65]. The primary antibodies used were as follows: Anti-vimentin (1:750; cat. no. sc-6260), anti-E-cadherin (1:500; cat. no. sc-597780), anti-N-cadherin (1:500; cat. no. sc-393933), anti-SHH (1:1000; Cell Signaling, cat. no. 2707), anti-GLI1 (1:000; Cell Signaling, cat. no. 3538), anti-β-actin (1:750; cat. no. sc-47778) and anti-GAPDH (1:750; cat. no. sc-32233). 

### 4.4. In Vitro Boyden Chamber Migration Assay

In vitro migration assay was conducted as described previously [66]. Briefly, parental and resistant cells were added to the upper chambers of the Boyden chamber containing DMEM with 1% FBS and were allowed to migrate towards 10% FBS in the lower chamber for 24 h. The migratory cells were stained with crystal violet solution for 10 min and photographed.

### 4.5. Colony Formation Assay

In vitro colony formation assay was carried out as described previously [67]. The parental and resistant cells were plated in duplicate at a density of 5000 cells per well of 6-well plate and allowed to grow for 10 days. The cells were then fixed and stained with crystal violet and photographed.

### 4.6. Wound Healing Assay

In vitro wound healing assay was performed according to our previous method [64]. Briefly, parental and resistant cells were grown in 12-well plate into a monolayer culture until 100% confluency. After scratching through the monolayer with a pipette tip, the media were replaced with fresh ones. The scratch closure was monitored and imaged at 0 and 24 h intervals. 

### 4.7. Quantitative Real-Time PCR (qRT-PCR)

Total RNA extraction and qRT-PCR were carried out as previously described [68]. Briefly, total RNA was extracted from parental and resistant cell lines using TRIzol (Invitrogen, Carlsbad, CA, USA) as per the manufacturer’s instructions. Taqman microRNA reverse transcription kit was used to prepare cDNA from total RNA. qRT-PCR for the quantitation of miR-506-3p was performed from cDNA products using TaqMan miRNA assays (Applied Biosystems, CA, Foster City, CA, USA) with specific primers and probes. To detect the expression level of different stem cell marker mRNA, cDNA was prepared using SuperScript II cDNA Synthesis kit (Thermo Fisher Scientific, Waltham, MA, USA) and qRT-PCR was performed using Taqman master mix (Applied Biosystems, Foster City, CA, USA) with cycling conditions of 95 °C for 10 min (followed by 95 °C for 15 s and 60 °C for 60 sec) for a total of 40 cycles. Taqman microRNA assay (Assay ID: 001050; Cat. no. 4427975) which detects mature miR-506-3p sequence (UAAGGCACCCUUCUGAGUAGA), the labeled primers, including Sonic Hedgehog (SHH; Hs00179843), POU5F1 (Hs00999632), ALDH1A1 (Hs00946916), KLF4 (Hs00358836), SOX2 (Hs04234836), NANOG (Hs02387400), CD44 (Hs05662929) and GAPDH (Hs02786624) were obtained from Applied Biosystems (Foster City, CA, USA). RNU6B and GAPDH were applied as internal controls for miR-506-3p and stem cell marker mRNA, respectively. The relative expression of miR-506-3p and different mRNAs were calculated by using the 2–∆∆CT method [69].

### 4.8. Apoptosis Assay

Apoptotic cell death was determined using cell death detection ELISA kits (Roche Diagnostic, Indianapolis, IN, USA) as we previously described [70]. Briefly, untreated and treated cells were harvested, lysed with lysis buffer, and the protein in the cytoplasmic supernatant was measured. Approximately 20 μL from the supernatant was added in the streptavidin-coated microplate and incubated with 80 μL of buffer mixture containing anti-histone-biotin and anti-DNA–peroxidase (POD) for 2 h with continuous shaking at room temperature. Microplates were washed with incubation buffer and the ABTS [2,2¢-azino-di-[3-ethylbenzthiazoline-6-sulfonic acid)) chromogen substrate was added to get a color. The reaction between POD and ABTS was photometrically determined using a microplate reader at 405 nm.

### 4.9. Spheroid Formation Assay

Spheroid formation assay was performed as our previous protocol [71] with little modification. Briefly, single-cell suspensions from pretreated cells were re-suspended at a density of 500 cells/mL in Mammocult media supplemented with 4 μg/mL heparin, and 0.48 μg/mL hydrocortisone (Stemcell Technologies, Vancouver, Canada) in ultra-low attachment 96-well spheroid microplates. Single-cell suspension cultures were allowed to grow for 8–10 days in the presence and absence of erlotinib with or without rhSHH protein (purchased from Peprotech, USA cat. no. 100-45) to form spheroids. The images of the spheroids were captured by an inverted microscope, and the size of the spheroids was measured by using NIS Element software.

### 4.10. Statistical Analysis

Data are represented as the mean  ±  SD. The statistical analysis was performed using the GraphPad Prism 6 (GraphPad Software, Inc., La Jolla, CA, USA) and PASS^15^ software, NCSS, tLLC (Kaysville, UT, USA). Means between the groups were calculated and compared among or within variants using an unpaired, two-sided Student *t* test. A *p* value of <0.05 was considered statistically significant. 

## Abbreviations

NSCLC	Non-small-cell lung cancer
EGFR	Epidermal growth factor receptor
TKI	Tryrosine kinase inhibitor
miRNA	microRNA
miR-506-3p	microRNA-506-3p
ER	Erlotinib resistance
SHH	Sonic Hedgehog
EMT	Epithelial–mesenchymal transition
rhSHH Pr.	Recombinant SHH protein
NGS	Next-generation sequencing
PFS	Progression-free survival
OS	Overall survival
